# Effects of packaging materials on storage quality of peanut kernels

**DOI:** 10.1371/journal.pone.0190377

**Published:** 2018-03-08

**Authors:** Xiaoji Fu, Shengping Xing, Huiwei Xiong, Hua Min, Xuejing Zhu, Jialin He, Jianxiong Feng, Honglei Mu

**Affiliations:** Institute of Food Science and Technology, Jiangxi Academy of Agricultural Sciences, Nanchang, Jiangxi,China; Oklahoma State University, UNITED STATES

## Abstract

In order to obtain optimum packaging materials for peanut kernels, the effects of four types of packaging materials on peanut storage quality (coat color, acid value, germination rate, relative damage, and prevention of aflatoxin contamination) were examined. The results showed that packaging materials had a major influence on peanut storage quality indexes. The color of the peanut seed coat packaged in the polyester/aluminum/polyamide/polyethylene (PET/AL/PA/PE) composite film bag did not change significantly during the storage period. Color deterioration was slower with polyamide/polyethylene (PA/PE) packaging materials than with polyethylene (PE) film bags and was slower in PE bags than in the woven bags. The use of PET/AL/PA/PE and PA/PE bags maintained peanut quality and freshness for more than one year and both package types resulted in better germination rates. There were significant differences between the four types of packaging materials in terms of controlling insect pests. The peanuts packaged in the highly permeable woven bags suffered serious invasion from insect pests, while both PET/AL/PA/PE and PA/PE bags effectively prevented insect infection. Peanuts stored in PET/AL/PA/PE and PA/PE bags were also better at preventing and controlling aflatoxin contamination.

## Introduction

Peanuts often suffer from oxidation and aflatoxin contamination during storage (Barros et al., 2003; Ding et al., 2014) [1,2]. Peanuts are usually harvested from late August until mid-October, depending on the particular season and the cultural practices in the south of China, and may need to be stored for a long period of time before they are sold. Peanuts are often stored under high humidity and high temperature conditions during the summer in the south of China, where daytime temperatures often exceed 35°C. The majority of the peanut crop is produced by smallholder farmers, who account for most of all of China's agricultural activity. Protecting harvested peanuts during storage is challenging for farmers, because insect pests, fungal growth, and lipid oxidation and rancidity can cause substantial losses in the mass and value of the grain after only a few months' storage [3–5]. A low temperature or air conditioned storehouse would be a useful post-harvest measure to maintain the quality and control aflatoxin production, but the majority of farmers are unable to adopt this recommendation because of the high application costs involved. Hermetic storage containers have been developed and distributed as a preferred method of grain storage for smallholder farmers. By severely restricting the flow of oxygen into the grain bulk, hermetic storage containers can reduce insect population growth during storage, preserve grain quality and inhibit aflatoxin production [6–8]. Such applications have been successful in effectively extending the shelf-life of maize, almond kernels and roast peanuts [8–11].

In China, some hermetic storage experiments with peanuts have been carried out; however, the application of different packaging materials for long-term storage of peanuts has not been studied. Therefore, there is little empirical evidence available on what are the most suitable packaging materials for storage of peanuts. The objectives of this study were to investigate the effects of different packaging materials on maintaining the quality of peanuts and inhibiting aflatoxin production, and to obtain suitable packaging materials for storing peanuts for different lengths of storage times.

## Methods

### Experimental design

The raw peanut kernels used in this study were produced in 2014 at Nanchang (Crop Institute Jiangxi Academy of Agricultural Sciences), and had an initial moisture content (m. c.) of 8%. The intact peanut kernels were placed in bags made from four different packaging materials: a) highly permeable woven bags, having an oxygen permeability of 5000 cm^2^/(m^2^ day atm) and a water vapor transmission rate of 660 g/(m^2^ day atm), b) polyethylene (PE) pouches, 75 μm in thickness, having an oxygen permeability of 600 cm^2^/(m^2^ day atm) and a water vapor transmission rate of 10 g/(m^2^ day atm), c) polyamide/polyethylene (PA/PE) pouches, 90 μm in thickness, having an oxygen permeability of 47.6 cm^2^/(m^2^ day atm) and a water vapor transmission rate of 3.9 g/(m^2^ day atm), and d) Polyester/aluminum/polyamide/polyethylene (PET/AL/PA/PE) pouches, 99 μm in thickness, having an oxygen permeability of 2.0 cm^2^/(m^2^ day atm) and a water vapor transmission rate of 1.0 g/(m^2^ day atm). For our experiments, the woven and PE bags were procured from the local market, and PA/PE and PET/AL/PA/PE bags were obtained from Yinfeng Plastic Color Printing Co. Ltd., Luoyang, China. The oxygen permeability and water vapor transmission rate were determined according to GB/T 19789–2005 [12] and GB/T 21529–2008 [13], respectively.

Ten kilograms of peanuts were packaged in each bag and the four packaging material types were replicated fifteen times (a total of 60 bags). Experiments were carried out during the period between December 2014 and December 2016. After 0, 6, 12, 18 and 24 months of storage, three separate identical samples (from three bags) were withdrawn from each packaging material type and subjected to chemical and sensory analysis. Samples were stored in Nanchang City Jiangxi Province of China, where the average temperature in summer is 27°C –34°C and the average relative humidity (r. h.) is 78.5%.

### Data collection

The following indicators of quality were measured during storage: peanut color, acid value, germination rate (%), and prevention of contamination with insects and aflatoxins.

#### Color measurement

The color of peanut kernels was measured using a Hunter Lab optical sensor colorimeter (model DP-9000, Hunter Associates Laboratory, Reston, VA, USA) and results expressed as color L* (lightness), a* (redness), and b* (yellowness) values. The results reported (L*, a* and b*) are the means of twenty determinations.

#### Measurement of acid value

The acid value was determined using a titration method, by measuring milligrams of potassium hydroxide (Yongda, Tianjin, China) used to neutralize 1 g of free fatty acids. The acid value was determined according to the official GBT5530-2005 [14] method for measurement of the acid value and acidity of animal and vegetable fats and oils.

#### Germination tests

Three samples of 100 seeds were removed from each packaging treatment. The seeds were bathed in a 1% bleach solution for 10 min and then rinsed three times under running tap water. Each sample was sowed in wet sand and placed in a climate incubator (28°C). After one week, the samples were removed and the number of seeds with at least part of the radical breaking through the seed coat was counted. Data was recorded as the percentage of the number of successfully germinated seeds out of the total number of peanuts sampled.

#### Insect damage

Each sample had three, 50 mL subsamples removed to evaluate the level of peanuts damage by insects. The number of damaged and undamaged kernels, by insects in each subsample, were visually counted and separated. Subsamples of each were dried to 0% moisture by heating in an oven at 60°C for 5 days. The dry weight of the damaged and undamaged peanuts for each subsample was then determined. Relative percent damage was calculated using the following equation as described by Alonso-Amelot et al. (2011) [15]:

$$X_{rel}=\left[\frac{\left(W_{u}*N_{d})-(W_{d}*N_{u}\right)}{W_{u}*(N_{u}+N_{d})}\right]*100$$

Nd = Number of damaged grains,
Nu = Number undamaged grains,
Wu = Dry weight of undamaged grains, and
Wd = Dry weight of damaged grains.

This equation compared the number of damaged and undamaged grains based on their weighted proportions. Using combined physical grain damage and weight took into account both visible insect damage and hidden damage caused by insect larvae feeding inside the peanuts. The equation is an effective alternative to estimate peanuts damage, which avoids collecting and weighing dust generated by insect feeding.

#### Determination of aflatoxin content

The aflatoxin content (AFB1) was determined with High Performance Liquid Chromatography (hplc) (Shimadzu, Tokyo, Japan) analysis, according to the official GB/T 5009.23–2006 [16] method for measurement of aflatoxins B1, B2, G1, and G2 in foods.

### Statistical analysis

Treatment condition effects on peanut coat color, acid value, germination rate (%), and prevention of insect and aflatoxin contamination were assessed using ANOVA. Post hoc comparisons were made between groups using LSD. Significant values were reported at the α = 0.01 level, unless noted otherwise. The ANOVA analyses included the time periods, but the statistical results are not shown in the figures or legends and tables.

## Results

### Lab values

**Fig 1:**
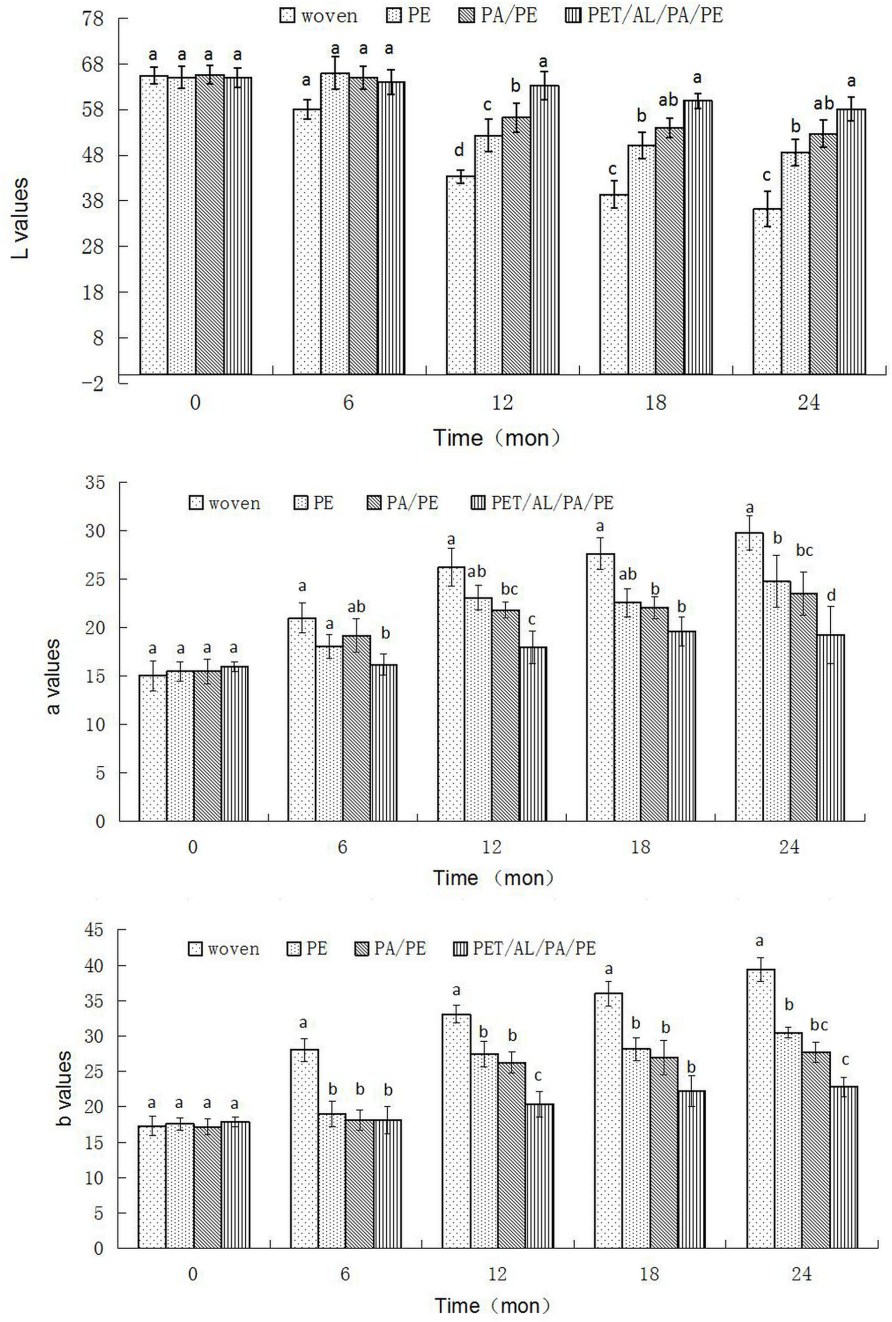
Effects of packaging treatments on parameters of peanuts during 24 months of storage. Storing peanuts in bags of different materials had a significant effect on seed coat color over time. The seed coat color of peanuts stored in PET/AL/PA/PE bags remained relatively stable during the 24 month study period; however, the samples packaged in wove, PE, and PA/PE pouches showed changes in all three color parameters (L*, a*, and b*) during storage. The L values of peanuts stored in woven bags decreased by 44.6%, and the a and b values increased by 97.6 and 128.0%, respectively, after 24 months. The L value of peanuts stored in PE decreased by 25.0% and the a and b values increased by 60.6% and 73.2%, respectively. The changes of Lab values for peanuts stored in PET/AL/PA/PE bags, in contrast, were relatively small after 24 months; the L value decreased only 10.5%, the a value increased 23.0%, and the b value increased 28.3%.

The seed color of samples in the woven bag (control) gradually changed color to dark red as storage time increased; L* parameters decreased significantly (F = 30.86; d. f. = 3,8; P<0.001) with a parallel increase in a* and b* values after 24 months of storage (Fig 1). Compared with the initial value (0 month), the samples packaged in PE (L*: F = 17.68; d. f. = 2,8; P = 0.003; a*: F = 19.09; d. f. = 2,8; P = 0.003; b*: F = 29.86; d. f. = 2,8; P = 0.001 and PA/PE (L*: F = 9.42; d. f. = 2,8; P = 0.014; a*:F = 10.47; d. f. = 2,8; P = 0.011; b*: F = 85.04; d. f. = 2,8; P<0.001 pouches showed statistically significant changes in all three color parameters after 12 months of storage. For samples stored in PET/AL/PA/PE pouches (light barrier bags), the color parameters did not change significantly compared to the initial values after 12 months of storage (L*: F = 0.336; d. f. = 2,8; P = 0.727; a*: F = 5.52; d. f. = 2,8; P = 0.044; b*: F = 2.03; d. f. = 2,8; P = 0.211). The most pronounced changes in peanut color were observed for peanuts packed in the woven and PE packages.

### Acid values

**Fig 2:**
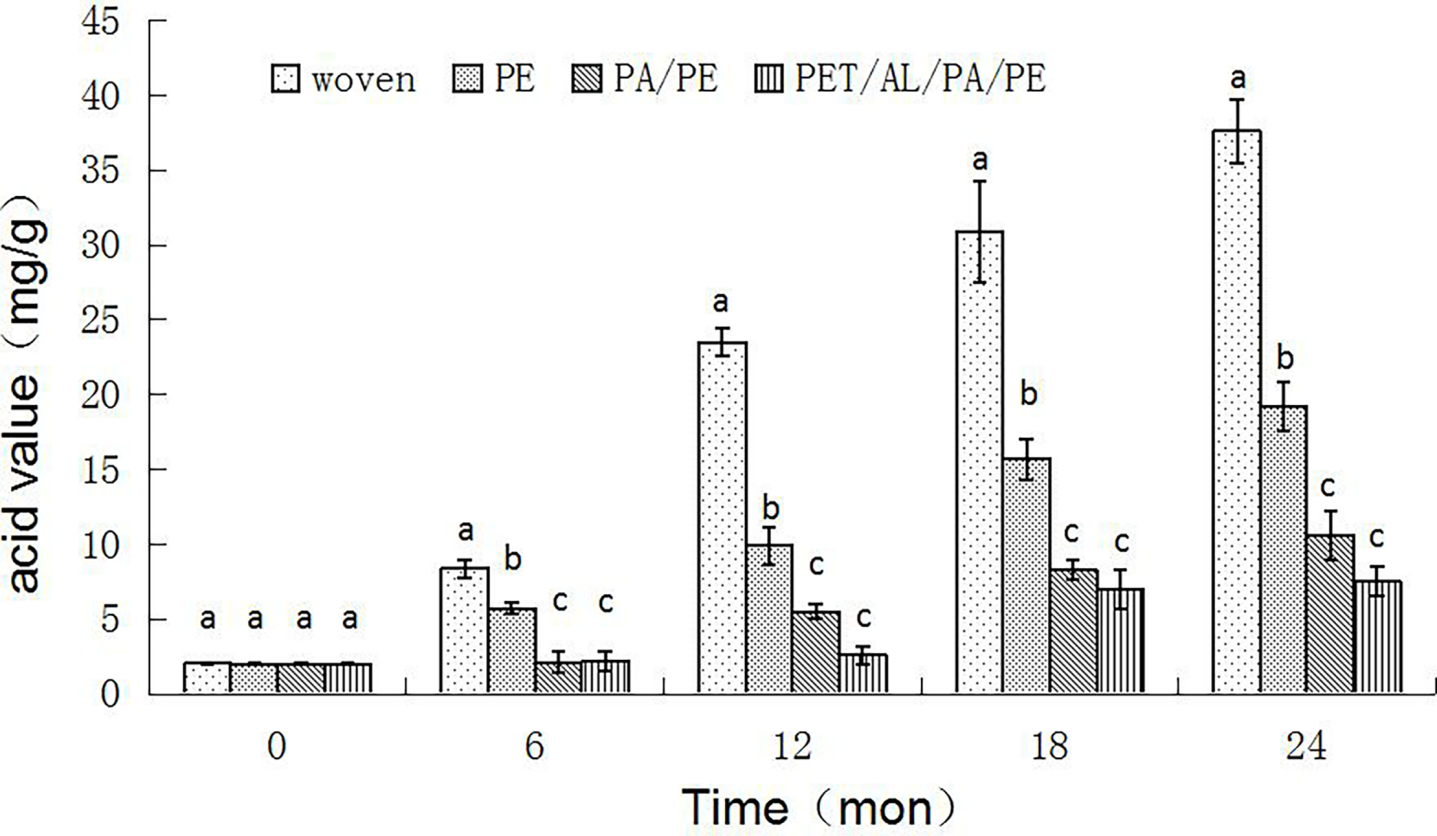
Effects of packaging treatments on acid values in peanuts during 24 months of storage. Acid values of the peanuts varied from month to month. Recorded levels of peanut acid values were highly variable from one time point to the next. The woven and PE bags showed a rapid increase, and the PA/PE and PET/AL/PA/PE bags showed a slow increase in acid values during the 24 months of storage.

The changes in acid values for samples during 24 months storage are shown in Fig 2. The initial acid values of the peanuts were very low, which is indicative of good product quality in terms of the degree of lipid oxidation. After 24 months of storage, peanuts packaged in the PET/AL/PA/PE pouches still had low acid values. The acid values of peanuts packaged in PE and PA/PE increased rapidly, while samples packaged in woven bags increased even further to reach levels of 30.9 mg/g. The results showed that good barrier properties of the packaging films resulted in better quality peanuts.

### Germination rates

Before conducting the trial (0 months), an average of 96.5 out of every 100 peanuts successfully germinated. The wove, PE, and PA/PE treatment groups showed a statistically significant decline in germination rates after 24 months of storage relative to initial values (wove: F = 890.13; d. f. = 4,14; *P*<0.001; PE: F = 248.77; d. f. = 4,14; *P*<0.001; PA/PE: F = 51.29; d. f. = 4,14; *P*<0.001). ANOVA comparison identified statistically significant differences among the four packaging treatments with regard to how much the treatments reduced peanut germination. The highest germination rates were observed in the PET/AL/PA/PE pouches (86 ± 5.3%; F = 2.79; d. f. = 4,14; *P* = 0.085), followed by the PA/PE pouches (51 ± 3.7%), the PE pouches (20 ± 5.1%), and finally the woven bags (0%) (Table 1).

**Table 1:** Results of ANOVA analyses comparing the effects of packaging treatments on germination rates in peanuts during storage.

Sampling period	Treatment	Germination rate (%)	Df	F-value	*P*-*value*
**0 months**	woven	97±2.5*	3,8	0.26	0.85
	PE	96±3.6*	3,8		
	PA/PE	98±2.3*	3,8		
	PET/AL/PA/PE	95±4.9*	3,8		
**6 months**	woven	84±2.8**	3,8	6.10	0.02
	PE	92±3.2*	3,8		
	PA/PE	93±5.2*	3,8		
	PET/AL/PA/PE	97±4.9*	3,8		
**12 months**	woven	45±3.0****	3,8	88.03	<0.001
	PE	56±2.2***	3,8		
	PA/PE	79±5.2**	3,8		
	PET/AL/PA/PE	93±4.9*	3,8		
**18 months**	woven	6±3.2****	3,8	188.54	<0.001
	PE	50±3.1***	3,8		
	PA/PE	66±6.2**	3,8		
	PET/AL/PA/PE	88±5.9*	3,8		
**24 months**	woven	0****	3,8	273.90	<0.001
	PE	20±5.1***	3,8		
	PA/PE	51±3.7**	3,8		
	PET/AL/PA/PE	86±5.3*	3,8		

*Asterisks indicate significant differences in germination rates (%) among the four packaging treatments. Treatments with the same number of asterisks are statistically similar and ones with different numbers are statistically different.

### Relative damage

**Fig 3:**
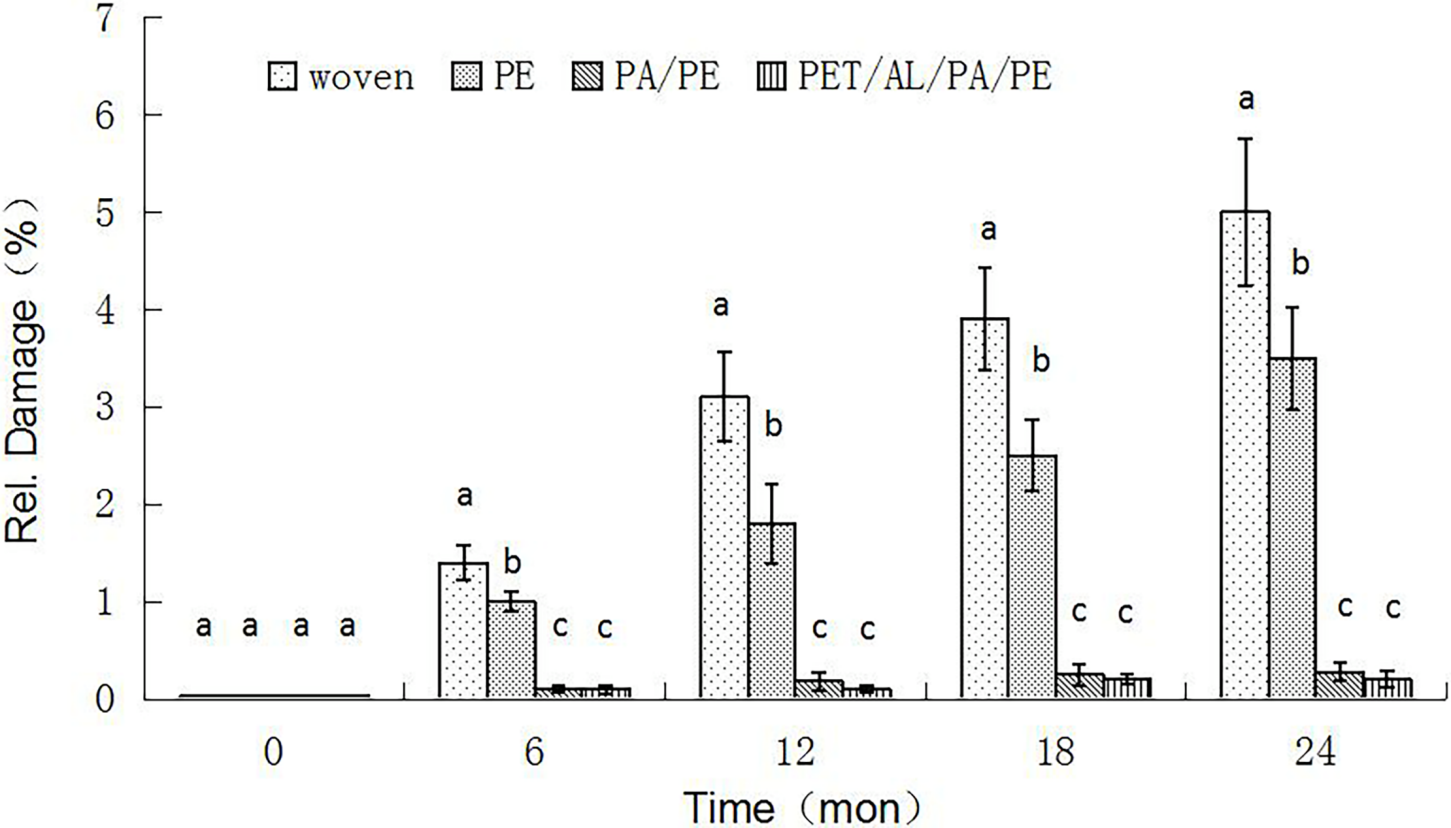
Effects of packaging treatments on relative damage to peanuts during 24 months of storage. Recorded levels of peanut damage were highly variable from one time point to the next in the woven and PE bags, which had an observed grain damaged of 5%, and the relative damage to peanuts in the PA/PE and PET/AL/PA/PE bags showed little increase during the 24 months of storage.

During storage, in addition to oxidation and rancidity, the peanuts can become easily damaged by insect pests. Insect damage to the stored peanuts varied greatly with time in our experiment (Fig 3). At the end of storage (24 months), the PA/PE composite film and PET/AL/PA/PE composite bags displayed good anti-pest properties, and there was no significant increase in peanut damage. However, the peanut damage of those stored in the woven and PE bags increased significantly (F = 141.52, d. f. = 3,8, *P<*0.001).

### Aflatoxin contamination

Table 2 shows that there were no aflatoxins detected in any of the packages, and there was no visible mildew on the surface of peanuts after 6 months of storage, because of the low temperatures used during the early stages of storage. However, after the summer period, aflatoxin B1 was detected in the woven bags, and aflatoxin B1 was detected in PE film bags (after 12 months). After 24 months of storage, aflatoxin contamination greatly exceeded the allowance for AFB1 in China (≤20 μg/kg) in the woven and PE film bags. There was white mildew (unidentified species) present in samples in the PA/PE film bags, but no aflatoxins were detected. In the PET/AL/PA/PE bags, there was no mildew or aflatoxins detected during the storage period.

**Table 2:** Results of ANOVA analyses comparing the effects of packaging treatments on aflatoxin contamination in peanuts during storage.

Sampling period	Treatment	AFB1 (μg/kg)	Df	F-value	*P*-*value*
**0 months**	woven	0*	3,8	-	-
	PE	0*	3,8		
	PA/PE	0*	3,8		
	PET/AL/PA/PE	0*	3,8		
**6 months**	woven	0*	3,8	-	-
	PE	0*	3,8		
	PA/PE	0*	3,8		
	PET/AL/PA/PE	0*	3,8		
**12 months**	woven	12.8±2.60*	3,8	52.26	<0.001
	PE	3.6±0.67**	3,8		
	PA/PE	0**	3,8		
	PET/AL/PA/PE	0**	3,8		
**18 months**	woven	55.7±5.61*	3,8	1095.55	<0.001
	PE	19.3±1.75**	3,8		
	PA/PE	0***	3,8		
	PET/AL/PA/PE	0***	3,8		
**24 months**	woven	101±7.32*	3,8	365.50	<0.001
	PE	39.4±5.74**	3,8		
	PA/PE	0***	3,8		
	PET/AL/PA/PE	0***	3,8		

*Asterisks indicate significant differences in aflatoxin contamination (μg/kg) among the four packaging treatments. Treatments with the same number of asterisks are statistically similar and ones with different numbers are statistically different.

## Discussion

In general, the presence of light and oxygen accelerated a decrease in L* parameters with a parallel increase in a* and b* parameters, resulting in a gradual darkening of product color during storage, which may be attributed to browning reactions due to oxidation of phenols [17]. Darkening of peanut kernels during storage has also been reported by several authors [3,18]. Ledbetter et al. [19] reported that exposure to light during storage was a highly significant factor in almond skin darkening, irrespective of almond variety. In case of storage under light (PE and PA/PE), darkening was more intense compared to that which occurred during storage in the dark (PET/AL/PA/PE pouches). These results show that the PET/AL/PA/PE pouches were the most suitable packages to maintain the original color of peanuts during storage.

The results showed that the germination rate decreased rapidly (in woven bags) during storage under a high temperature and high humidity environment, even when the seed had been dried to a safe m. c. Storing peanut in the sealed foil bag had no effect on either the grain's m. c. or germination potential, and these values remained stable even after 12 months of storage. For tropical regions, using a barrier against water vapor transmission would prevent stored seed from absorbing water when humidity is high [20], which would encourage seed deterioration. The grain stored in bags with good barrier properties had good germination rates, due to low oxygen and humidity conditions inside the bags [21]. The results showed that the germination rates of peanuts stored in PET/AL/PA/PE bags were the highest, followed by peanuts in the PA/PE bags, which were higher than those in PE and woven bags. Therefore, the better the barrier properties of packages, the higher the germination rates of peanuts in this experiment. Low oxygen and humidity conditions within the bags also maintained good quality characteristics, and the peanuts that were stored in PA/PE or foil bags were found to still have low acid values after 24 months of storage.

When the oxygen concentration reaches 8%, the number of pests will be severely inhibited [22], and our experiments gave similar results. Both PA/PE and PET/AL/PA/PE bags had low oxygen concentrations in packages (PA/PE <7.48%; PET/AL/PA/PE <5.16%), and no pests were found in these bags. Woven bags had significantly higher (*P*<0.01) levels of pest contamination in comparison to the other three packaging materials. The results showed that pest breeding was significantly affected by the barrier property of the packaging materials (*P*<0.01).

Williams et al. [8] reported that both the spread of *A*. *flavus* and aflatoxin accumulation in moist maize can be controlled by placing the grain in a Purdue Improved Crop Storage bag. When the oxygen concentration is reduced to 5%, aflatoxin production is severely inhibited [23]. Our results indicated that the PA/PE and PET/AL/PA/PE bags with high barrier properties (low oxygen environment) can control aflatoxin production. Peanuts stored in woven and PE film bags suffered aflatoxin contamination, even when the initial raw material contained the safe m. c. [24] showed that an 8% m. c. was high enough to support fungal growth in peanuts, and *A*. *flavus* could grow and accumulate aflatoxin rapidly if exposed to a r. h. of 60%. Pore space r. h. in the woven and PE bags could increase to 60% r. h. easily in the south of China, because of the low barrier properties.

In summary, this study examined the effects of four types of packaging materials on peanut quality, including peanut color, acid value, germination rate and prevention of insect and aflatoxin contamination. The results showed that the packaging materials had a notable influence on peanut storage quality indexes. Compared to woven and PE film bags, PA/PE and PET/AL/PA/PE bags with high barrier properties (moisture and oxygen barrier) can inhibit oxidative discoloration of the seed coat, prevent pest contamination and control aflatoxin production.

Different storage bags can maintain the quality of peanuts for different lengths of time; therefore it is important to choose the correct packaging to store peanuts. The selection of peanut packaging materials can be determined according to the length of storage time and quality required. For peanut kernels that are to be used for only short-term storage (i.e. not more than six months), general PE bags can be used. If peanuts are to be stored for one year, good barrier properties of PA/PE composite film and PET/AL/PA/PE bags are recommended. If appearance and quality requirements are higher, and if the storage time is longer than one year, the strong barrier properties of PET/AL/PA/PE bags are required. Based on the present data, the optimum packages for storage of peanut kernels for a long period of time were the PET/AL/PA/PE pouches. When PET/AL/PA/PE pouches were stored at room temperature the product shelf-life was maintained for 24 months.

## References

[1] Barros G, Torres A, Palacio G, Chulze S. Aspergillus species from section Flavi isolated from soil at planting and harvest time in peanut-growing regions of Argentina. J. Sci. Food Agric.2003;83: 1303–1307.

[2] Ding X, Wu L, Li P. et al Risk Assessment on Dietary Exposure to Aflatoxin B1in Post-Harvest Peanuts in the Yangtze River Ecological Region. Toxins. 2014;7(10):4157–4174. 10.3390/toxins7104157 PMC4626727 26501322

[3] Chen H, Xiong LR, Wang J. Influence of packaging materials on storage property of peanut under normal temperature. Transactions of the CSAE. 2012;28(3): 269–273. (in Chinese with English abstract)

[4] Boxall RA. Damage and loss caused by the larger grain borer. Integr. Pest Manag. 2002; Rev. 7: 105–121.

[5] Sheikh AS, Hirata T, Ishitani T. Quality preservation of peanuts by means of plastic packaging. Pakistan Journal of Scientific and Industrial Research.1985;28(1): 46–51.

[6] Baoua IB, Margam V, Amadou L, Murdock LL. Performance of triple bagging hermetic technology for postharvest storage of cowpea grain in Niger. J. Stored Prod. Res. 2012;51(2): 81–85.

[7] Gao X, Cai JP, Huang SX, et al Study on the microbe activity of different packaging peanut. Cereals and Oils Processing. 2009;5: 47–50. (in Chinese with English abstract)

[8] Williams SB, Baributsa D, Woloshuk C. Assessing Purdue Improved Crop Storage (PICS) bags to mitigate fungal growth and aflatoxin contamination. Journal of Stored Products Research, 2014; 59:190–196.

[9] García-Pascual P, Mateos M, Cardonell V, Salazar D. Influence of storage conditions on the quality of shelled and roasted almonds. Biosystems Engineering.2003;84(2): 201–209.

[10] Severini C, Gomes T, Pilli T, Romani S, Massini R. Autoxidation of packed almonds as affected by Maillard reaction volatile compounds derived from roasting. Journal of Agriculture and Food Chemistry. 2000;48(10): 4635–4640. 10.1021/jf0000575 11052711

[11] Severini C, Pilli T, Baiano A. Auto oxidation of packed roasted almonds as affected by two packaging films. Journal of Food Processing and Preservation.2003;27(4): 321–335.

[12] General Administration of Quality Supervision, Inspection and Quarantine of the People’s Republic of China. GB/T 19789–2005 Packaging material-Test method for oxygen gas permeability characteristics of plastic film and sheeting Coulome tricsensor China standards Press 2005;2–6

[13] General Administration of Quality Supervision, Inspection and Quarantine of the People’s Republic of China. GB/T 21529–2008 Determination of water vapour transmission rate for plastic film and sheeting—Electrolytic detection sensor method China standards Press 2008;2–4

[14] General Administration of Quality Supervision, Inspection and Quarantine of the People’s Republic of China.GBT5530-2005 Animal and vegetable fats and oils-Determination of acid value and acidity China standards Press 2005;1–6.

[15] Alonso-Amelot, Miguel E, Avila-Núnez JL. Comparison of seven methods for stored cereal losses to insects for their application in rural conditions. J. Stored Prod. Res.2011; 47 (2): 82–87.

[16] Ministry of Health, People's Republic of China. GB/T 5009.23–2006 Determination of aflatoxins B1,B2,G1,G2 in foods China standards Press 2006;5–10.

[17] Ryan D, Robards K. Phenolic compounds in olives. Analyst.1998;123: 31R–44R.9581017

[18] Fu XJ, Min H, Zhang Q, He JL, Zhu XJ, Feng JX. Study on Gas Filling Packaging Technology of Peeled Peanut, Journal of Peanut Science. 2012;41(4):26–29.(in Chinese with English abstract)

[19] Ledbetter C, Palmquist D. Degradation of almond pellicle color coordinates at different storage temperature. Postharvest Biology and Technology.2006;40(3): 295–300.

[20] Devereau AD, Myhara R, Anderson C. Chapter 3: Physical factors in postharvest quality In: Golob PP., Farrell G, Orchard JE. (Eds.), Crop Post-Harvest: Science and Technology: Principles and Practice. Blackwell Science Ltd, Ames, Iowa, pp.2002; 62–92.

[21] Scott BW, Larry LM, Dieudonne B. Safe storage of maize in alternative hermetic containers. Journal of Stored Products Research 2017;71: 125–129.

[22] Deng YX, Zhao ZM, Li LS. Research advance of environmental factors on stored grain insects[J].Grain Storage.2003;1:5–12.

[23] Diener UL, Davis ND. Aflatoxin Formation in Peanuts by Aspergillus flavus. Agricultural Experiment Station/Auburn University, Auburn, Ala 1977.

[24] Zhang CS, Zhao Q, Feng JX, Sun J, Yu LN, Bi J, et al Study on the key environmental factors aspergillus flavus growth and aflatoxin production. Applied Mechanics & Materials, 2014; 668–669: 1550–1553.

